# Social, behavioral and biological correlates of cardiorespiratory fitness according to sex, nutritional status and maturity status among adolescents. A cross-sectional study

**DOI:** 10.1590/1516-3180.2017.0405190218

**Published:** 2018-06-25

**Authors:** André Oliveira Werneck, Danilo Rodrigues Silva, Ricardo Ribeiro Agostinete, Rômulo Araújo Fernandes, Enio Ricardo Vaz Ronque, Edilson Serpeloni Cyrino

**Affiliations:** I Undergraduate Student, Centro de Educação Física e Esporte (CEFE), Universidade Estadual de Londrina (UEL), Londrina (PR), Brazil.; II PhD. Professor, Department of Physical Education, Universidade Federal de Sergipe (UFS), São Cristóvão (SE), Brazil.; III Doctoral Student, Department of Physical Education, Universidade Estadual Paulista Julio de Mesquita Filho, São Paulo (SP), Brazil.; IV PhD. Professor, Department of Physical Education, Universidade Estadual Paulista Julio de Mesquita Filho, São Paulo (SP), Brazil.; V PhD. Professor, Centro de Ciências da Saude (CCS), Universidade Estadual de Londrina (UEL), Londrina (PR), Brazil.; VI PhD. Professor, Centro de Ciências da Saude (CCS), Universidade Estadual de Londrina (UEL), Londrina (PR), Brazil.

**Keywords:** Physical fitness, Adolescent health, Motor activity, Social class

## Abstract

**BACKGROUND:**

Our aim was to analyze multilevel correlates of cardiorespiratory fitness (CRF) according to sex, nutritional status and maturity status among adolescents.

**DESIGN AND SETTING:**

Cross-sectional study conducted in public schools.

**METHODS:**

This was a cross-sectional study on 1,209 adolescents aged between 10 and 17 years. CRF was estimated from the 20-meter shuttle run test. Anthropometric data on body mass index and waist circumference were obtained. Somatic maturation was assessed from the peak height velocity. Questionnaires were used to evaluate socioeconomic variables (family income, parents’ education level and number of siblings) and behavioral variables (physical activity, screen time and alcohol and tobacco consumption) among the parents and adolescents.

**RESULTS:**

Boys, adolescents with normal weight and on-time maturers presented greater CRF (P < 0.05). Concerning socioeconomic correlates, girls (tobacco smoking, alcohol consumption, physical activity and screen time), adolescents with normal weight (alcohol consumption, physical activity and screen time), and on-time maturers (alcohol consumption, physical activity during childhood and habitual physical activity) demonstrated higher numbers of behavioral correlates with CRF. Normal-weight adolescents presented a higher number of biological correlates (chronological age, age at peak height velocity and waist circumference).

**CONCLUSIONS:**

Different correlates were observed according to sex, nutritional status and somatic maturation status. However, habitual physical activity, waist circumference and chronological age seemed to be the strongest factors associated with cardiorespiratory fitness among adolescents.

## INTRODUCTION

Cardiorespiratory fitness measured during physical effort is the ability to provide oxygen effectively to skeletal muscles,^1^ which is recognized as a protective factor against obesity,^2^ cardiovascular risk^3^^,^^4^ and mortality.^5^ This capacity seems to be active from early ages to adulthood,^6^ thus highlighting the importance of promoting adequate levels of this physical fitness component from infancy onwards. Therefore, understanding possible factors associated with cardiorespiratory fitness (i.e. correlates) among children and adolescents is relevant for development of effective interventions to improve this factor.

Variables that correlate with cardiorespiratory fitness during adolescence can be grouped into different “levels” based on their proximity to cardiorespiratory fitness. Firstly, there are socioeconomic and parental environmental factors, such as socioeconomic status, parental educational status and physical activity relating to cardiorespiratory fitness.^7^ In addition, there are behavioral associated factors, such as physical activity, sedentary behavior and others.^7^ Finally, close to cardiorespiratory fitness, there are biological associated factors such as fatness^2^ and chronological age.^8^

Specifically regarding biological factors, it can be expected that cardiorespiratory fitness will increase over the growth process.^9^ The rate of growth (biological maturation) can influence cardiorespiratory fitness directly, through development of cardiovascular and respiratory systems,^10^ as well as having an important role in several psychological and behavioral factors.^11^ Thus, biological maturation seems to be a key correlate during adolescence. Moreover, body composition is also strongly associated with cardiorespiratory fitness in this age group^12^ and may confound association results if not considered.^13^

Our aim in the present study was to analyze social, behavioral and biological correlates of cardiorespiratory fitness according to sex, nutritional status and maturity status, through a multilevel approach, among Brazilian adolescents. The initial hypothesis of the study was that sex, nutritional status and maturity status would be strongly associated with cardiorespiratory fitness^3^ and would have a role in several of its associated factors at different levels. These would include biological factors (e.g. waist circumference), behavioral factors (e.g. tobacco smoking, alcohol consumption, physical activity and screen time) and social factors (e.g. socioeconomic status, parental variables and number of siblings). In this manner, different correlates of physical activity would exist according to these biological constructs (boys versus girls; normal weight versus overweight/obese; and late versus on-time versus early maturity).

## METHODS

### Sample

This was a cross-sectional study conducted among Brazilian adolescents aged between 10 and 17 years who were enrolled in public schools. The city of Londrina, Paraná, where the study was conducted, has a medium human development index.^14^ The current study forms part of a project entitled “Prevalence of metabolic syndrome and cardiovascular risk factors in adolescents from Londrina” for which the sample size calculation was based on the following parameters: prevalence of metabolic syndrome of 4%; α of 0.05; margin of error of two percentage points; and design effect of 2.0. The sample size was further increased by 20% to compensate for any participant withdrawals. Following the recommendation of Luiz and Magnanini,^14^ at least 900 adolescents should be recruited.

Sample recruitment was performed in two stages. First, all public schools in the city were separated into regions (north, south, east, west and central) and two schools were randomly selected from each region. Subsequently, two or three classes were randomly identified in each school and all students within these classes (except those using prescription medicine or undergoing treatment for an illness) were invited to participate in the study. Students failing to return a consent form signed by their parents were considered ineligible. The total number of adolescents recruited was 1,395; however, due to missing data, especially parental data, the final sample was composed of 1,209 adolescents (549 boys and 660 girls).

Reassuringly, it was demonstrated that these participants with complete data were representative of all the adolescents who were initially enrolled in the study.^4^ Moreover, all the questionnaires were reapplied to a subsample of 129 adolescents from the pilot study, who were not included in the final sample of this article, with the aim of calculating intraclass coefficients.

The local ethics committee approved all the study procedures, which adhered to the principles of the Declaration of Helsinki (procedural number 10655/2012).

### Cardiorespiratory fitness

The 20-meter shuttle run test^15^ was administered in sports courts and was used to define maximal aerobic speed and consequently to estimate cardiorespiratory fitness. Subsequently, peak oxygen consumption, in ml.kg^-1^.min^-1^, was estimated according to the equation 1 proposed by Leger et al.:^16^

$${VO}_{2}\;peak\;=\;31.025\;+\;3.238*S\;-\;3.248*A\;+\;0.1536*S*A$$ (1)

Where:

S: = final speed (kmh^-1^);
A: = age (years).

### Biological maturation and nutritional status

Biological maturation was estimated through the somatic maturation method derived from the estimated age at peak height velocity, as proposed by Mirwald et al.^17^ This method estimates distance in years from peak height velocity through anthropometric variables (height, seated height, leg length and body mass). Predictions for age at peak height velocity are determined through subtracting the maturity offset from chronological age. For categorization, we used the method of standard deviations (sd) derived from the sample (late: > +1 sd; on time: +/- 1 sd; early: < 1 sd).

Maturity offset for boys (in years) = -9.236

- + [0.0002708 * (leg length * seated height)]
- + [-0.001663 * (age * leg length)]
- + [0.007216 * (age * seated height)]
- + [0.02292 * (body mass/height *100)]

Maturity offset for girls (in years) = -9.376

- + [0.0001882 * (leg length * seated height)]
- + [0.0022 * (age * leg length)]
- + [0.005841* (age * seated height)]
- + [0.002658 * (age * body mass)]
- + [0.07693 * (body mass/height *100)]

### Body mass index and waist circumference

Body mass was accessed using a digital scale (Balmak; precision = 0.1 kg) and height was measured using a stadiometer with precision of 0.01 cm), in accordance with standardized procedures in the literature. ^18^ Nutritional status was estimated though the body mass index (BMI) using the formula: body mass (kg)/height (m)², with the following values for technical errors of measurement: weight = 0.68% and height = 0.37%. Waist circumference was measured between the rib cage and the iliac crest (minimum circumference), as recommended in the literature,^19^ using an anthropometric tape with a precision of 0.1 cm. All the procedures were performed while the adolescent subjects were wearing light clothing.

### Parental/socioeconomic factors

Data on the parents’ tobacco smoking and alcohol consumption were collected through a self-report questionnaire, with dichotomous questions (yes or no), which were answered by the parents of the adolescents. We made the following assumptions regarding the data:

1. We considered the parents to be alcohol consumers if they reported using alcohol at least once a week in answer to the question: “How often do you drink alcoholic drinks?”
2. Similarly, we considered the parents to be tobacco smokers if they reported using tobacco in answer to the question: “Do you have a tobacco smoking habit?”
3. Physical activity levels were self-reported using the Baecke questionnaire, which captures information regarding exercise practice and duration of practice. In line with physical activity guidelines,^20^ parents who reported being physically active for more than 180 minutes/week were classified as active.
4. Maternal and paternal BMI were calculated using self-reported values for weight and height in response to questionnaires that were answered by each parent. Previous studies had reported good validity of self-reported body size data among Brazilian adults.^21^

### Behavioral factors

The behavioral factors were defined as follows:

1. Information on alcohol consumption and tobacco smoking among the adolescents was obtained in a dichotomous manner (yes or no), through indication of the use during the previous 30 days. For quality control, the intraclass correlation coefficients (ICCs) for a repeated pilot sample for alcohol consumption and tobacco smoking were respectively 0.74 and 0.78.
2. The Baecke questionnaire ^22^ was applied to estimate habitual physical activity and the score proposed by the authors of the original study were taken to be an indicator of habitual physical activity (ICC = 0.73).
3. Physical activity during early life was estimated through a question asking about sports practice during childhood. This question is used in the literature.^23^^,^^24^
4. Adolescents who reported doing systematized exercise (practicing a structured program of exercise under supervision from a coach) for at least one year were considered to have been active during childhood.
5. Screen time (ICC = 0.77) in minutes per week and at the weekend was taken to be an indicator of sedentary behavior.

### Statistical analysis 

Frequencies, means and standard deviations were used to compare groups, along with the Kruskal-Wallis test (for somatic maturity status) and the Mann-Whitney test (regarding sex and nutritional status). Partial correlations (adjusted according to sex) between three levels of correlates and cardiorespiratory fitness were used. The correlation coefficients were interpreted as follows: trivial (r < 0.1), small (0.1 > r < 0.3), moderate (0.3 > r < 0.5), large (0.5 > r < 0.7), very large (0.7 > r < 0.9), nearly perfect (r > 0.9) and perfect (r = 1). To test the association between correlates and cardiorespiratory fitness, hierarchical linear regression was conducted as described by Victora et al.,^25^ with three blocks of variables that were entered in the following order:

1. parental and socioeconomic-related variables;
2. behavioral variables; and
3. biological variables.

All analyses were performed using the SPSS software (version 23.0) with a significance level of P < 0.05.

## RESULTS

**Figure 1:**
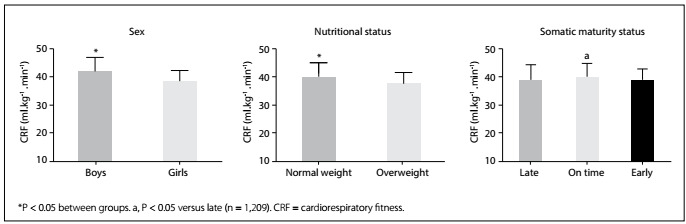
Cardiorespiratory fitness according to sex, nutritional status and somatic maturity status.

Boys presented greater habitual physical activity and screen time than girls (P < 0.001). Moreover, regarding biological variables, boys, normal-weight adolescents and late-maturing adolescents presented higher means for chronological age, age at peak height velocity and waist circumference (Table 1). On-time maturing adolescents presented higher cardiorespiratory fitness than late-maturing adolescents (P = 0.027) **(**Figure 1**)**.

**Table 1:** Characteristics of the sample according to hierarchical levels, sex, nutritional status and somatic maturity status

	Sex		Nutritional status		Somatic maturity status		
	Boys (n = 549)	Girls (n = 660)	Normal weight (n = 958)	Overweight/obese (n = 251)	Late (n = 168)	On time (n = 871)	Early (n = 170)
Chronological age (years)	13.0 ± 1.5*	12.8 ± 1.4	12.9 ± 1.5*	12.6 ± 1.4	14.3 ± 1.4*	12.8 ± 1.3	12.0 ± 1.1
Age at peak height velocity (years)	14.4 ± 0.7*	12.4 ± 0.7	13.4 ± 1.2*	12.8 ± 1.2	14.4 ± 1.0*	13.3 ± 1.1	12.2 ± 1.0
Waist circumference (cm)	67.7 ± 9.0*	65.5 ± 7.9	63.4 ± 5.3*	78.2 ± 8.2	64.3 ± 6.3	65.5 ± 7.8	73.8 ± 10.1^†^
Tobacco smoking (%)	8.4%	8.2%	7.6%	8.5%	15.5%*	6.9%	4.5%
Alcohol consumption (%)	18.8%	17.9%	18.1%	15.8%	23.2%*	17.4%	12.8%
Early physical activity (%)	74.0%*	60.0%	65.4%	70.2%	62.4%	67.5%	64.8%
Habitual physical activity (score)	8.5 ± 1.4*	7.7 ± 1.3	8.1 ± 1.4	8.0 ± 1.5	8.0 ± 1.5	8.1 ± 1.4	7.9 ± 1.4
Screen time (hours)	8.7 ± 5.8*	7.7 ± 4.7	8.2 ± 5.1	8.1 ± 4.9	8.1 ± 4.9	8.2 ± 5.4	7.7 ± 4.7
Socioeconomic status (low) (%)	65.5%	68.3%	67.3%	66.4%	73.8%	65.7%	67.4%
Maternal educational status¹ (%)	33.8%	24.3%	26.4%	25.2%	19.0%	27.3%	27.6%
Paternal educational status¹ (%)	32.4%	25.8%	27.8%	29.4%	21.9%	29.1%	29.4%
Maternal tobacco smoking (%)	14.1%	16.0%	15.6%	12.5%	15.0%	15.7%	11.0%
Paternal tobacco smoking (%)	27.3%	29.7%	28.6%	27.6%	30.0%	28.5%	26.0%
Maternal alcohol consumption (%)	21.7%	25.1%	24.7%	18.4%	33.0%	24.5%	18.0%
Paternal alcohol consumption (%)	52.1%	53.8%	55.1%	55.4%	60.0%	53.0%	46.0%
Maternal body mass index (kg/m²)	26.7 ± 5.5	26.4 ± 5.2	26.3 ± 5.3	27.6 ± 5.4	25.9 ± 5.3	26.6 ± 5.3	27.0 ± 5.5
Paternal body mass index (kg/m²)	26.5 ± 4.6	26.3 ± 4.3	26.1 ± 4.1*	27.3 ± 4.9	26.4 ± 3.8	26.1 ± 4.3	27.8 ± 4.9^†^
Maternal physical activity (%)	4.3%	2.5%	3.0%	3.9%	5.0%	2.7%	4.0%
Paternal physical activity (%)	7.2%	6.8%	6.5%	7.9%	4.0%	7.3%	7.0%
Number of siblings (n)	2.5 ± 1.9	2.6 ± 2.1	2.6 ± 2.0	2.2 ± 1.8	2.9 ± 2.0	2.5 ± 1.9	2.4 ± 2.0

*P < 0.05 for trend or differences between all groups. ^†^denotes significant difference compared to “on time” in somatic maturity status.

Partial correlations between associated factors (hierarchically divided into three levels, i.e. parental and socioeconomic-related variables, behavioral variables and biological variables) and cardiorespiratory fitness according to sex, nutritional status and somatic maturity status are presented in Table 2.

**Table 2:** Partial correlations between independent variables and cardiorespiratory fitness according to sex, nutritional status and maturity status (n = 1,209)

	Sex		Nutritional status		Somatic maturity status		
	Boys	Girls	Normal weight	Overweight/obese	Late	On time	Early
First level							
Socioeconomic status	-0.017	-0.092	0.077	0.065	-0.126	-0.007	-0.072
Maternal educational status	-0.108	-0.025	-0.020	-0.090	0.006	-0.055	-0.073
Paternal educational status	-0.084	-0.052	-0.004	-0.037	0.058	-0.039	-0.068
Maternal tobacco smoking	0.074	0.041	0.039	0.019	0.090	0.017	-0.073
Paternal tobacco smoking	0.005	0.059	0.040	-0.107	-0.045	0.012	-0.068
Maternal alcohol consumption	0.031	0.007	-0.013	0.014	0.073	-0.012	-0.015
Paternal alcohol consumption	0.015	0.033	0.005	0.129	0.140	0.026	-0.006
Maternal body mass index	0.008	-0.056	0.049	-0.141	-0.050	0.007	-0.047
Paternal body mass index	-0.103	-0.074	0.004	-0.209	0.028	-0.079	-0.052
Maternal physical activity status	0.046	0.097	0.052	0.144	0.154	0.045	0.040
Paternal physical activity status	0.006	0.020	0.026	0.033	0.070	0.029	-0.082
Number of siblings	0.106	0.067	0.077	-0.062	0.164	0.051	0.151
Second level							
Tobacco smoking	0.022	-0.180	-0.049	-0.153	-0.198	0.001	-0.055
Alcohol consumption	-0.044	-0.118	-0.068	-0.078	-0.098	-0.065	0.110
Early physical activity	0.123	0.080	0.174	0.158	0.221	0.144	0.102
Habitual physical activity	0.224	0.180	0.323	0.215	0.369	0.286	0.326
Screen time	-0.016	-0.158	-0.047	0.039	-0.082	-0.032	0.065
Third level							
Chronological age	­-0.172	-0.533	-0.325	-0.360	-0.351	-0.287	-0.072
Age at peak height velocity	0.021	-0.237	0.281	0.034	0.419	0.349	0.309
Waist circumference	-0.403	-0.356	-0.130	-0.250	-0.246	-0.307	-0.460

The values in bold indicate P < 0.05. Adjusted according to sex.

Among boys, their mother’s educational status (low magnitude), their physical activity levels (low magnitude) and waist circumference (moderate magnitude) were related to cardiorespiratory fitness. On the other hand, girls with lower socioeconomic status (trivial magnitude), whose mothers were active (trivial magnitude), did not smoke tobacco (small magnitude) or drink alcohol (small magnitude), and who had higher habitual physical activity (small magnitude) and lower screen time (small magnitude), presented higher cardiorespiratory fitness. Regarding peak height velocity, girls who were more advanced in maturation presented higher cardiorespiratory fitness (small magnitude). The adolescents of both sexes with smaller waist circumference presented higher cardiorespiratory fitness (moderate magnitude) **(**Table 2**)**.

Concerning associations according to nutritional status, normal-weight and overweight/obese adolescents presented positive correlations between early and habitual physical activity and cardiorespiratory fitness. Conversely, both groups also presented negative correlations between chronological age and waist circumference and cardiorespiratory fitness. In the specific analysis on normal-weight adolescents, higher cardiorespiratory fitness was associated with socioeconomic status (trivial correlation), more siblings (trivial correlation), not drinking alcohol (trivial correlation) and age at peak height velocity (small correlation), while among overweight/obese adolescents, higher cardiorespiratory fitness was associated with lower paternal nutritional status (small correlation) and non-consumption of tobacco (small correlation) **(**Table 2**)**.

When divided according to somatic maturity status, cardiorespiratory fitness in all three groups was positively associated with habitual physical activity and age at peak height velocity and negatively associated with waist circumference. In late-maturing adolescents, cardiorespiratory fitness was associated with early physical activity (small correlation), lower chronological age (moderate correlation), not smoking tobacco (small correlation) and number of siblings (small correlation). On the other hand, in on-time maturing adolescents, cardiorespiratory fitness was associated with early physical activity (small correlation) and lower chronological age (small correlation) **(**Table 2**)**. Table 3 summarizes the linear regression (significant variables) with the objective of obtaining possible correlates.

**Table 3:** Hierarchical regression models of correlates of cardiorespiratory fitness according to sex, nutritional status and maturity status (n = 1,209)

	Sex		Nutritional status		Somatic maturity status		
	Boys	Girls	Normal weight	Overweight/obese	Late	On time	Early
First level							
Adjusted R²	0.012	0.015	0.216	0.095	0.263	-	0.112
Socioeconomic status	-	-0.34 (-0.63 to -0.04)	-	-	-	-	-
Maternal educational status	-0.46 (-0.87 to -0.04)	-	-	-	-	-	-
Paternal body mass index	-	-	-	-0.14 (-0.268 to -0.029)	-	-	-
Maternal physical activity status	-	2.38 (0.41 to 4.34)	-	-	4.57 (0.686 to 8.462)	-	-
Number of siblings	-	-	0.195 (0.058 to 0.331)	-	-	-	0.297 (0.040 to 0.555)
Second level							
Adjusted R²	0.081	0.104	0.280	0.149	0.307	0.203	0.165
Tobacco smoking	-	-2.65 (-4.12 to -1.18)	-	-2.92 (-5.494 to -0.354)	-	-	-
Alcohol consumption	-	-0.92 (-1.83 to -0.01)	-1.372 (-2.115 to -0.629)	-	-	-1.249 (-2.049 to -0.449)	-
Early physical activity	-	-	-	1.51 (0.278 to 2.749)	-	0.671 (0.011 to 1.330)	-
Habitual physical activity	0.93 (0.59 to 1.27)	0.44 (0.18 to 0.71)	0.705 (0.492 to 0.918)	-	1.10 (1.499 to 1.703)	0.515 (0.278 to 0.752)	0.619 (0.207 to 1.032)
Screen time	-	-0.14 (-0.21 to -0.07)	-0.099 (-0.155 to -0.044)	-	-	-	-
Third level							
Adjusted R²	0.246	0.333	0.401	0.253	0.458	0.365	0.425
Chronological age	-	-1.54 (-1.80 to -1.28)	-0.854 (-1.134 to -0.574)	-0.548 (-0.987 to -0.108)	-1.53 (-2.085 to -0.994)	-0.545 (-0.771 to -0.319)	-
Age at peak height velocity	-	0.70 (0.19 to 1.21)	-0.675 (-1.230 to -0.121)	-	-	-	-
Waist circumference	-0.22 (-0.27 to -0.17)	-	-0.080 (-0.150 to -0.010)	-0.120 (-0.191 to -0.050)	-	-0.199 (-0.239 to -0.159)	-0.192 (-0.238 to -0.146)

Non-significant models are not presented; P < 0.05; Adjusted according to sex.

## DISCUSSION

Our aim was to analyze correlates of cardiorespiratory fitness in three levels (parental and socioeconomic-related variables, behavioral variables and biological variables) according to sex, nutritional status and somatic maturity status. To our knowledge, this is the first study that considered this range of associated factors in three levels stratified according to sex, nutritional status and maturity status. The main findings of the present study indicate that there are correlates of cardiorespiratory fitness at different levels, and that there are different correlates according to sex, nutritional status and maturity status. Greater numbers of correlates were observed among girls, normal-weight adolescents and on-time maturing adolescents. Furthermore, the correlates that appeared more frequently among the models were habitual physical activity, waist circumference and chronological age.

Boys tend to present higher values for cardiorespiratory fitness because of their biological and behavioral characteristics, especially after the biological maturation process, since boys have greater muscle mass, which is associated with cardiorespiratory fitness.^2^^,^^9^^,^^26^ Moreover, boys have higher levels of physical activity than girls, which is related to cardiorespiratory fitness.^7^ This finding corroborates other studies and can also be explained by the built environment, in which boys receive greater incentive to practice physical activity.^27^^,^^28^ Similarly, nutritional status has a role in estimating cardiorespiratory fitness, such that because of the growth process, cardiorespiratory fitness seems be underestimated among overweight/obese adolescents.^16^ Concerning biological maturation, it can be expected that cardiorespiratory fitness will increase over the course of the maturation process, also due to changes in muscle mass and evolution in organic systems.^8^

We found that only the mother’s educational status, number of siblings, habitual physical activity and waist circumference were associated with cardiorespiratory fitness among boys. On the other hand, the number of associated factors was higher among girls: these were mainly behavioral correlates (habitual physical activity, tobacco smoking, alcohol consumption and screen time). This could be because cardiorespiratory fitness is more affected by biological variables in boys than in girls,^2^ while female cardiorespiratory fitness is more affected by behavioral variables. Moreover, the correlates among boys were stronger and, consequently, fewer than among girls, thus indicating that a greater range of possible interventions aiming towards improvement of cardiorespiratory fitness exists for girls, while more specific correlates are necessary among boys.

A similar point of view can be applied to our results relating to nutritional status. Normal-weight adolescents presented higher values for cardiorespiratory fitness and higher numbers of significant correlates than did overweight/obese adolescents. This could be due to the relationship between adiposity and cardiorespiratory fitness,^30^ which presented a strong relationship in our models for overweight/obese adolescents and overlapped with other correlates.

Furthermore, late-maturing adolescents presented only maternal physical activity, habitual physical activity and chronological age as correlates; on-time adolescents presented a broad range of behavioral correlates; and early maturers presented significant correlates with the number of siblings, habitual physical activity and waist circumference. Given our hypothesis, correlates may change between maturity categories because of their intrinsic biological and psychological alterations.^11^ Chronological age probably entered the late-maturing model due to the lack of more advanced maturation of systems.^10^ On the other hand, age did not enter the early-maturing model because the prevalence of obesity among early-maturing adolescents is high and therefore waist circumference would seem to be a better predictor of cardiorespiratory fitness.

In general, we found correlates of cardiorespiratory fitness among all three theoretical levels. In the first level, cardiorespiratory fitness was inversely related to two indicators of socioeconomic status (maternal educational level and socioeconomic status). This phenomenon seems to occur especially through the possible influence of socioeconomic status on some behaviors,^31^ consequently influencing cardiorespiratory fitness. In addition, we found that home environmental correlates were also associated with cardiorespiratory fitness (paternal nutritional status, maternal physical activity levels and number of siblings), which would indicate possible social influence and transfer of behaviors between people who live together.^32^

Although a large part of cardiorespiratory fitness can be explained biologically,^33^ healthy behaviors also have an effect. The most consistent association that we found was with physical activity, which has a clear effect, especially in the exercise domain.^34^ Contrary to the well-recognized beneficial effects of physical exercise, we also found that drinking alcohol and smoking tobacco had an adverse effect on cardiorespiratory fitness, which is consistent, given their deleterious effects on health.^35^ Moreover, early physical activity can have a particular role, even genetically.^36^ Screen time is also a negative form of behavior, especially because of the lack of muscle contractions. In addition to the independent effects of each of these behaviors, they may interact, given that they are correlated.^37^

Biological factors are closer to the outcome, given that this is also biological.^7^ In this regard, we found that cardiorespiratory fitness increased with chronological age. This occurs because several biological developmental processes occur during growth.^9^ Similarly, biological maturation affects cardiorespiratory fitness, although it can influence both biological and behavioral correlates.^11^ Although biologically early-maturing adolescents may present higher physical fitness, they also have greater likelihood of presenting unhealthy behaviors^11^^,^^38^ that can influence health outcomes.

The three levels of correlates indicate different types of interventions, as well as risk groups. According to our results, older adolescents, girls and adolescents with greater waist circumference should receive special attention. Moreover, family-based interventions and behavioral interventions among adolescents appear to be interesting interventions for increasing cardiorespiratory fitness^33^^,^^39^

Our study presents some limitations that need to be acknowledged. Firstly, our measurement of parental characteristics was through self-reporting and may have presented bias, although self-reports among adults have demonstrated good validity.^20^ Moreover, both physical activity and early physical activity were estimated through questionnaires, which despite the possibility of presenting bias, showed good reproducibility (ICC = 0.73). On the other hand, we used an indirect, but valid method of estimation of cardiorespiratory fitness that has been widely used in the literature.^3^^,^^7^^,^^8^^,^^14^ Finally, because of our study design (cross-sectional), we cannot indicate the causality between exposures and outcome.

## CONCLUSIONS

In summary, we confirmed that there are correlates of cardiorespiratory fitness at different levels of variables (social, behavioral and biological). Among the associated factors tested, habitual physical activity, waist circumference and chronological age seem to be strongly associated with cardiorespiratory fitness, even though different correlates were found according to sex, nutritional status and somatic maturation status.
